# Optimization of the Preparation Parameters of High-Strength Nickel Layers by Electrodeposition on Mild Steel Substrates

**DOI:** 10.3390/ma14185461

**Published:** 2021-09-21

**Authors:** Dongai Wang, Feihui Li, Yan Shi, Meihua Liu, Bin Liu, Qing Chang

**Affiliations:** 1 Department of Mechanics, Tianjin University of Commerce, Tianjin 300134, China; wangda@tjcu.edu.cn (D.W.); shiyan@tjcu.edu.cn (Y.S.); liubin@tjcu.edu.cn (B.L.); changqing@tjcu.edu.cn (Q.C.); 2 School of Biotechnology and Food Science, Tianjin University of Commerce, Tianjin 300072, China; tjlifeihui@tjcu.edu.cn

**Keywords:** electroplated nickel, orthogonal test, mechanical properties, residual stress, watts plating solution

## Abstract

The electrodeposition process parameters were optimized for the acquisition of high-strength monolithic nickel layers on Q235A substrates based on the Watts nickel plating solution using the DC electrodeposition method. Based on the study of the electrochemical polarization behavior of nickel ions in Watts’ plating solution, 16 experimental protocols were selected according to the orthogonal test method. The residual stress, microhardness, modulus of elasticity, and surface roughness of the nickel plating were tested by X-ray diffractometer, nano-mechanical test system, and surface profilometer, respectively, to investigate the influence of current density, temperature, and PH on the mechanical properties of nickel plating, so as to determine the best process solution for the preparation of high-strength nickel plating. The results of the study show that the mechanical properties of the nickel deposits electrodeposited onto Q235A are optimized when plating at a current density of 3 A/dm^2^, a bath temperature of 45 °C, and a pH of 3.5. The nickel-plated layer has a minimum grain size of 34.8 nm, a microhardness of 3.86 GPa, a modulus of elasticity of 238 GPa, and a surface roughness Ra of 0.182 μm.

## 1. Introduction

Q235A is a widely used low-carbon steel with good all-around performance due to its strength, plasticity, and welding properties, and it is widely used in construction and engineering structures as well as in the production of mechanical parts with low performance requirements and other unimportant abrasive parts [1]. However, Q235A has a low wear resistance, and its chemical stability in the natural environment is also low and not corrosion-resistant. This largely limits its application in areas where mechanical strength is required. In contrast, nickel metal has high chemical stability, good plasticity, and calendering, and nickel plating has high hardness as well as wear and corrosion resistance, with good surface properties. Therefore, it is widely used in surface engineering in machinery, instrumentation, automotive, and other industries. The electrodeposition of nickel plating, with high productivity, simple equipment, low cost, and other characteristics, has been commonly used in practical engineering applications. However, there are still some problems with the preparation of nickel plating by electrodeposition. In fact, this process has size and surface effects when the grain size reaches the nanoscale, resulting in properties that differ significantly from those of the bulk material; moreover, the crystal structure and size of the plating can also vary with process parameters during electrodeposition, thus affecting its mechanical properties [2]. The microhardness and the modulus of elasticity of nickel plating differ from those of the bulk material, and they vary with parameters such as current density, plating bath temperature, and pH. In addition, due to thermal effects and lattice mismatch between the coating and the substrate as well as hydrogen precipitation during the electrodeposition process, residual stresses can be generated inside the coating, which can promote the sprouting of cracks or accelerate their expansion, thus leading to coating failure [3]. The traditional alkaline battery shell material is made of low-carbon steel plated with nickel to improve its corrosion resistance [4]. The battery casing is prepared by nickel plating the low-carbon steel strip and then deep punching the strip with the nickel plating on the surface in order to directly obtain the nickel-plated battery casing. This production process has the advantages of having high efficiency, a fast rate, and low cost, but it is important to ensure that the nickel coating is intact, without cracks, wrinkles, or even peeling. The ideal nickel coating has high strength, toughness, hardness, and corrosion resistance, and can better improve the wear resistance and corrosion resistance of the surface layer of the substrate. Therefore, it is important to prepare a nickel coating with excellent mechanical properties by optimizing the process parameters.

There are many factors that affect the appearance and performance of nickel plating, including the pre-plating treatment of the substrate [5], the composition of the electrolyte [6], current density [7,8,9], plating solution temperature [10], pH [11], agitation [12], and additives [13], among others. Nan Zhou et al. [4] investigated the effect of DC plating and pulse plating on the surface morphology and corrosion resistance of the plated layers during the electrodeposition of pure nickel plating on Q235A substrates. Jiang Yanping and others [14] studied the hardness and elastic modulus of a Q235A surface electroplated with nickel before and after heat treatment by the nanoindentation technique. But there is a lack of systematic studies on the correlation between the mechanical properties and the preparation process parameters of electrodeposited nickel coatings on Q235A substrates. This paper investigated the influence of plating process parameters on the mechanical properties of nickel plating prepared on mild steel Q235A substrates through orthogonal tests; it also established the correlation between the microhardness, elastic modulus, residual stress, and surface roughness of the nickel plating prepared, and the preparation process parameters, so as to obtain the optimal preparation process. The study provides technical support for the preparation of high-strength nickel plating on Q235A substrates of mild steel.

## 2. Experimental Preparation

### 2.1. Substrate Specification and Pre-Treatment

**Figure 1:**
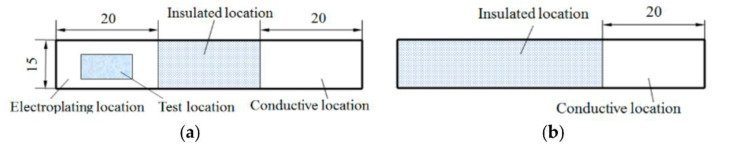
Q235A substrate specification diagram: (**a**) Electroplated surface and (**b**) nonelectroplated surface [15].

The substrate specification was 60 mm × 10 mm × 3 mm, with a plating area of 20 mm × 10 mm. The non-plated areas of the sample was sealed with insulating material, as shown in Figure 1. The surface of the sample plating area was first ground roughly with corundum grinding wheels of grit size 60# and 80#, and then polished with sponge wheels of grit size 300# and 800#. Eventually, the surface roughness parameter of each sample (Ra = 0.2 μm) was measured using a surface profilometer. The specific process flow is as follows: rough grinding with corundum grinding wheels—fine grinding with sponge wheel—measurement of surface roughness parameter *Ra*—distilled water rinsing—chemical degreasing—distilled water rinse—electrochemical degreasing—distilled water rinse—nickel plating—distilled water rinse—blow drying, and final testing of plating related parameters.

### 2.2. Electroplating Solution Components and Electrochemical Test for the Redox Behavior of Nickel Ions in a Plating Solution

**Figure 2:**
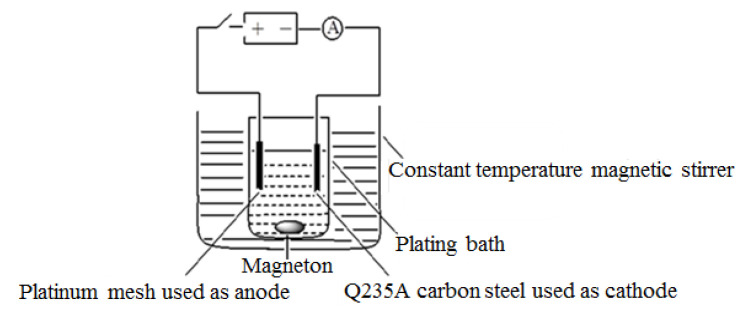
Schematic diagram of the DC electrochemical deposition device.

The chemical material used in the electroplating solution was analytically pure and provided by Tianjin Guangfu Fine Chemical Research Institute in Tianjin, China. The composition and content of the plating solution were respectively: (1) NiSO_4_-6H_2_O, 240 g/L; (2) NiCl_2_-6H_2_O, 20 g/L; (3) H_3_BO_3_, 20 g/L. The direct current nickel plating apparatus is shown in Figure 2.

**Figure 3:**
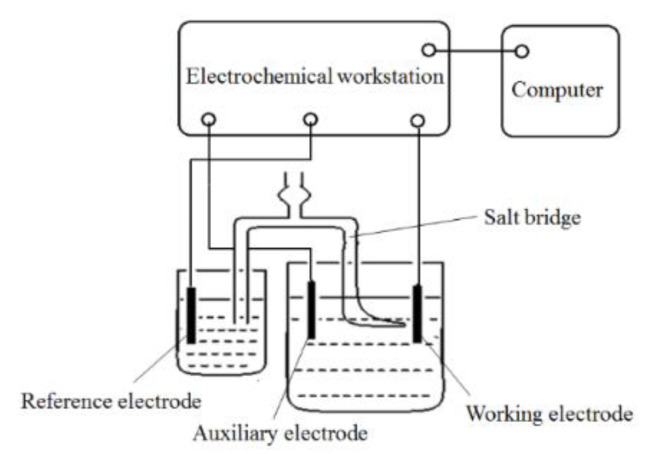
Diagram of the electrochemical test circuit and system.

A three-electrode and two-loop system was used to test the redox behavior of nickel ions in the plating solution. A Luggin capillary salt bridge was prepared using agar powder C_12_H_18_O_9_ and potassium nitrate KNO_3_. A three-electrode system, with Q235A as the working electrode, platinum as the auxiliary electrode, and saturated calomel electrode (SCE) as the reference electrode, was established to test the electrochemical behavior of Ni^2+^ with an electrochemical workstation CHI660E, Beijing Chinese science days Technology Co., Ltd., Beijing, China and a computer, as shown in Figure 3. On the basis of this test, a reasonable range of temperature and pH of the plating solution for nickel plating was determined.

Based on electrochemical tests on the redox behavior of nickel ions in plating solutions, the plating process parameters were selected in the following ranges [16,17]: (1) cathode current density 1.5~3 A/dm^2^; (2) pH 3.5~5; (3) temperature 45 °C~60 °C; (4) stirring speed 100 r/min; (5) plating time 70 min. The specific experimental protocol was designed with the use of the orthogonal test method, using the L_16_ (3^4^) orthogonal table for the plating solution temperature, pH value, and current density; the levels of each factor are shown in Table 1.

### 2.3. Testing

The crystal structure and residual stress of the plating were analyzed using a D/MAX-2500 X-ray diffractometer, Tokyo, Japan. The test conditions were: copper target, tube pressure of 40 Kv, scanning rate of 8°/min. The residual stress was measured on a grain surface of (400) with lateral inclination angles of 0°, 15°, 25°, 35°, and 45°, respectively. The hardness and elastic modulus of the plating were tested using a NanoIndenter XP Test System, Minneapolis, Minnesota, USA. The parameters used for the test included the diamond Berkovich indenter, loading and unloading rates of 40 nm/s, the press depth of 2500 nm, and the press was continued for 10 s at the maximum press depth. As the layer thickness prepared here was 25 μm and the press depth was 10% of the plating thickness, the mechanical properties of the measured plating were not affected by the substrate [18]. The surface roughness parameter Ra of the plating was tested using a 2302A Surface Profile Measuring Instrument manufactured by Harbin Gauge & Cutting Tools Group, Harbin, China using the following parameters: sampling length of 2.5 mm, assessment length of 12.5 mm, and drive box glide speed of 0.5 mm/s.

## 3. Results and Discussion

### 3.1. Analysis of the Electrochemical Behavior of Nickel Ions in the Plating Solution

#### 3.1.1. Effect of Plating Solution Temperature on the Electrochemical Behavior of Nickel Ions

**Figure 4:**
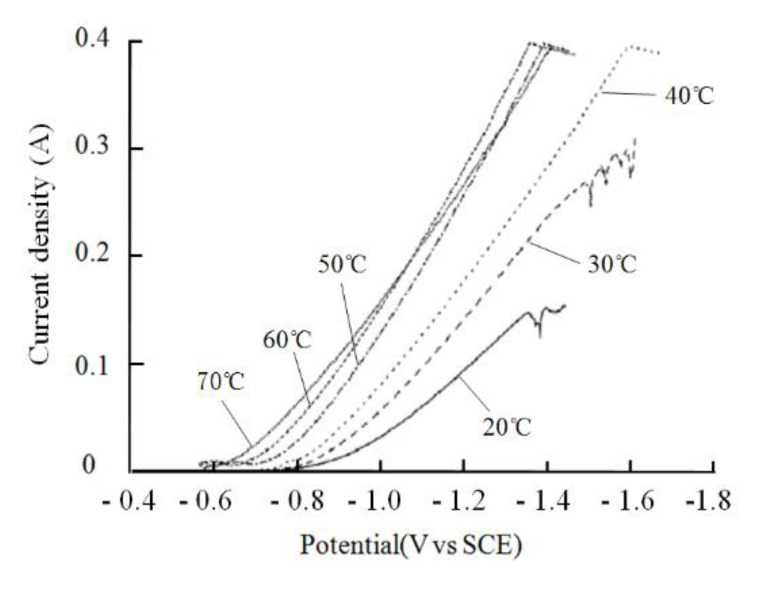
Nickel ion cathodic polarization curves in the plating solution at different temperatures.

The cathodic polarization curves measured by the constant potential method at different temperatures are shown in Figure 4, starting from the open circuit potential and scanning at 1 mV/s. According to Figure 4, the cathodic polarization in the plating solution basically decreased with increasing temperature, which was consistent with the theoretical analysis. When the voltage was between −0.57 V and −0.7 V, the nickel deposition current was low at different temperatures in the plating solution, and the lower the temperature of the bath, the lower the deposition current. When the voltage was negative up to −0.7 V, the nickel deposition current increased as the bath temperature rose. This is due to the fact that the potential does not reach the potential at which nickel deposition begins under low overpotential conditions, which means that the significant reduction precipitation reaction of nickel has not yet begun. Therefore the current density is relatively low. When the potential shifted negatively and the overpotential reached a certain value, the nickel reduction reaction began and the current rose significantly. Increasing the temperature in the plating solution reduces the viscosity of the solution, which can intensify the thermal movement of the ions in the solution, facilitating the transport of ions to the cathode surface, increasing the rate of mass transfer in the liquid phase, reducing the concentration polarization, and increasing the current at the same overpotential. When the temperature of the plating solution exceeded 50 °C, the cathode current density increased as the temperature of the plating solution increased, which accelerated the deposition of the cathode nickel layer. However, too high a temperature of the plating solution would also accelerate the evaporation of the plating solution. According to the test results, a reasonable bath temperature for nickel plating on mild steel surfaces is between 40 °C and 60 °C.

#### 3.1.2. Effect of pH of the Plating Solution on the Electrochemical Behavior of Nickel Ions

**Figure 5:**
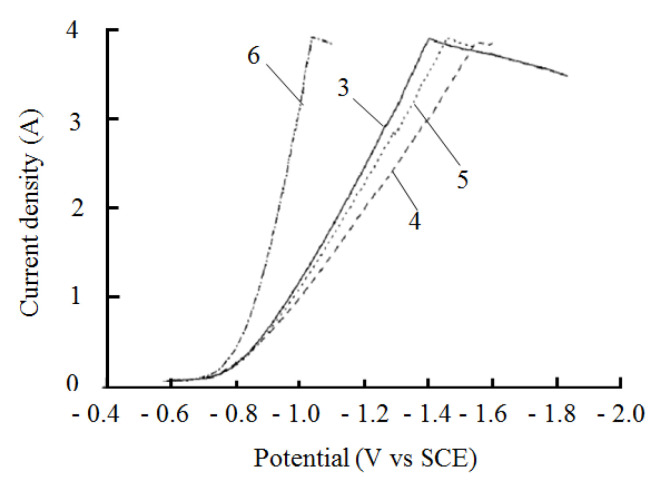
Cathodic polarization curves of nickel ions at different pH values in the plating solution.

Figure 5 shows the cathodic polarization curves for different plating solution pH values. When the pH in the plating solution increased from 3.0 to 4.0, cathodic polarization was enhanced. When the pH value in the plating solution was 3, the solution became more acidic and a massive reduction of hydrogen inhibited the deposition of nickel ions. Increasing the pH value in the plating solution increased the cathodic overpotential and increased the cathodic current efficiency. By continuing to increase the pH to 6.0, the ions OH^−^ & and Ni^2+^ in the plating solution generated Ni(OH)_2_ impurities, which affected the quality of the coating. Based on the analysis above, the pH value of the plating solution was reasonably chosen to be between 3.0 and 5.0.

Combining the current densities shown in Figure 4 and Figure 5, it was found that when the current density was below 1 A/dm^2^, the overvoltage was relatively low and the cathodic polarization was small. When the current density exceeded 1 A/dm^2^, the concentrated polarization in the plating solution affected the electrodeposition process. As the cathodic overpotential increased, the current density tended to flatten out. When the current density was between 3 A/dm^2^ and 4 A/dm^2^, the cathodic potential continued to shift negatively and the polarization curve appeared to plateau, indicating that the concentrated polarization in the plating solution had been formed, the Ni^2+^ in the plating solution was depleted, and the mass transfer process was hindered, with the hydrogen precipitation occurring during the deposition process. This led to a reduction in the quality of the nickel plating. In summary, the current density chosen for the experiments here is between 1 A/dm^2^ and 3 A/dm^2^.

### 3.2. Effect of Plating Process Parameters on the Mechanical Properties of the Coating

#### 3.2.1. Effect on the Microhardness of the Plating

**Figure 6:**
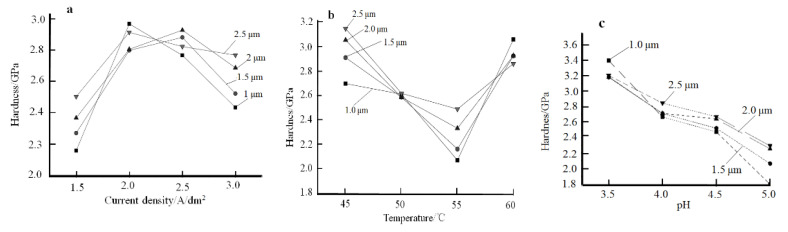
Effect of plating process parameters on the hardness of the layer: (**a**) Current density; (**b**) Temperature of the plating solution; (**c**) pH.

The effect of current density, bath temperature, and pH value on the microhardness of nickel plating is shown in Figure 6. The hardness of the plating was tested using the nanoindentation method, with four selected depths: 1 μm, 1.5 μm, 2 μm, and 2.5 μm. According to Figure 6a, as the cathodic current density increased from 1.5 A/dm^2^ to 3.0 A/dm^2^ during electroplating, the microhardness of nickel plating tended to increase and then decrease, which was in accordance with the classical electrocrystallization theory. The process of electrodepositing nickel plating comprises two stages. The first stage involves the transport of ions from the electrolyte to the electrode surface and their discharge. The second stage involves the entry of atoms into the lattice and the growth of crystals. According to the theory of nucleation kinetics for thin-film growth, the relationship between the rate of nucleation W and the overpotential η_k_ is as follows [19]:

(1) $$W=B\exp(-b/\eta^{2}{}_{k})$$

where B and b are constants. According to the formula above, the higher the overpotential during plating, the higher the chance of nucleation of the coating, and the higher the number of nuclei formed. As a result, the coating organisation becomes denser due to the smaller size of the nuclei in the coating, resulting in an increase in the hardness of the coating. Thus, as the current density increases during plating, the cathodic polarization increases accordingly, so that the electrodeposition process takes place at a higher potential. This increases the driving force of the crystal-shaped nucleus. As a result, a large number of nucleations are favored and a fine-grained nickel coating is thereby obtained during the electrodeposition process. This is accompanied by a gradual increase in the microhardness of the plating. However, when the current density continues to increase during the plating process, the cathode reacts too quickly and causes the phenomenon of differential polarization. At the same time, the amount of hydrogen precipitation increases, which leads to a reduction in the denseness of the layer and therefore a reduction in its microhardness.

The effects of the plating solution temperature on the microhardness of the plated layer is shown in Figure 6b. According to Figure 6b, when the temperature of the plating solution increases from 45 °C to 60 °C during plating, the hardness of the plated layer shows a pattern that first decreases and then increases. The influence of the bath temperature during plating on the hardness of the coating is mainly related to the cathode overpotential [18]. The hardness of the plating shows a tendency to fall and then rise in relation to the dual effect of the temperature of the plating solution on cathodic polarization during plating. According to the Arrhenius equation, the relationship between the reaction rate constant k and the temperature of the plating solution is as follows:

k = A exp (−E/RT)(2)

where k is the positive reaction rate constant. A is the frequency factor and E is an energetic quantity. For a given reaction, A and E are constants greater than zero. R is the gas constant and T is the absolute temperature of the plating solution. In electrodeposition, the temperature of the plating solution has a dual effect on the electrodeposition cathodic process. When other conditions remain the same, the temperature of the plating solution increases, cathodic polarization decreases, and the crystallization of the layer is coarser. This is because when the temperature of the plating solution increases, the diffusion rate of the ions in the plating solution increases. The end result is a reduction in differential concentration polarization in the plating solution. At the same time, according to the Arrhenius equation, there is a faster dehydration of hydrated nickel ions and hydrated hydrogen ions during the plating process, with the temperature of the plating solution increasing, resulting in an increase in the rate of reductive stress. This enhances the activity of the metal ions on the cathode surface, and produces significantly lower polarization and easier electrodeposition reactions. As a result, the microhardness of the plating is reduced. However, the activity of reactive ions in the electrolyte increases with a rise in the temperature of the plating solution, thus increasing the ultimate current density, which in turn increases the polarization effect. As a result, the microhardness of the plated layer appeared to decrease first and then increase as the temperature of the plating solution went from low to high. This result is consistent with the results obtained by Xu Jiaojiao [20].

The pattern of the effect of pH on the microhardness of the plating is shown in Figure 6c. The microhardness of the plating decreases with increasing pH of the plating solution. This is due to the fact that as the pH of the plating solution increases, the concentration of ionic OH^−^ on the electrode surface increases, leading to the formation of Ni(OH)_2_ between the cathode and the diffusion layer and adsorption onto the cathode surface, thus preventing the effective deposition of nickel ions. The reactions of Ni(OH)_2_ are entrapped in the coating, forming spongy deposits that affect the quality of the coating and lowering its microhardness.

**Figure 7:**
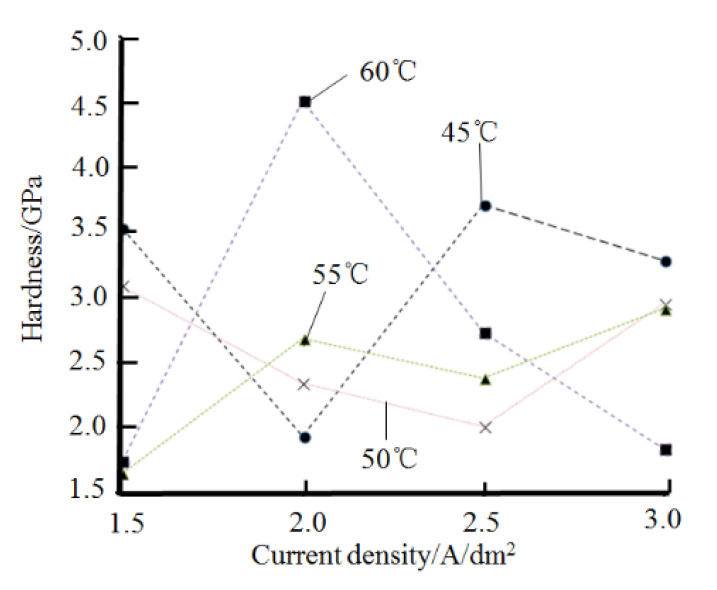
Relationship between current density, plating bath temperature, and hardness of the plated layer.

Figure 7 shows the relationship between current density, bath temperature, and the microhardness of the coating. The preliminary optimized plating process parameters for the preparation of high-hardness nickel plating by electrodeposition are obtained as follows: (1) current density at 2.0 A/dm^2^, plating solution temperature at 60 °C, pH 3.5; (2) current density at 2.5 A/dm^2^, temperature at 45 °C, pH 3.5; (3) current density at 3.0 A/dm^2^, temperature at 45 °C, pH 3.5.

#### 3.2.2. Effect on the Elastic Modulus of the Plated Layer

**Figure 8:**
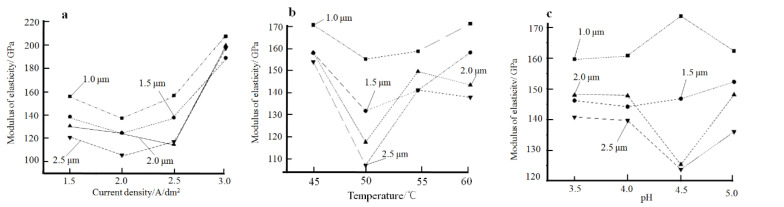
Effect of plating parameters on the elastic modulus of the plated layer: (**a**) current density, (**b**) temperature of the plating solution, (**c**) pH.

Figure 8 shows the pattern of influence of plating parameters on the elastic modulus of the coating. Here, the hardness of the plating is tested using the nanoindentation method, with four selected depths: 1 μm, 1.5 μm, 2 μm, and 2.5 μm.

According to Figure 8a, when the current density is increased from 1.5 A/dm^2^ to 2 A/dm^2^, the modulus of elasticity of the plating decreases slightly. This may be due to the increased current density, which accelerates the rate of nickel ion transport, resulting in insufficient time for the nickel ions to migrate to the pores of the plating. This leads to an increase in amounts of voids in the plating. As the current density continues to increase, the selective orientation changes in the plating, the texture coefficients along the crystal plane (111) increase, and the (111) crystal plane selective orientation gradually rises [21]. Due to the anisotropic nature of the nickel plating, the modulus of elasticity of the various crystalline surfaces of the nickel plating is different. Frederick Milstein’s research concluded that [22] the modulus of elasticity of the nickel plated along crystal faces (111), (110), and (100) are E_111_ = 302.6 GPa, E_110_ = 233.5 GPa, and E_100_ = 138.5 GPa, respectively. As a result, the modulus of elasticity of the coating increased with the increase in the texture coefficients of the plated nickel along crystal surface (111).

The effect of the temperature of the plating solution on the modulus of elasticity of the plated layer is shown in Figure 8b. As the temperature of the plating solution increases, the modulus of elasticity of the nickel plating shows a general trend of first decreasing and then increasing. On the one hand, as the temperature of the plating solution increases, the cathodic polarization decreases, the grain size of the plated layer increases, while the grain boundaries decrease. The number of dislocations within the grains of the nickel-plated layer that hinder the movement of dislocations is reduced.

**Figure 9:**
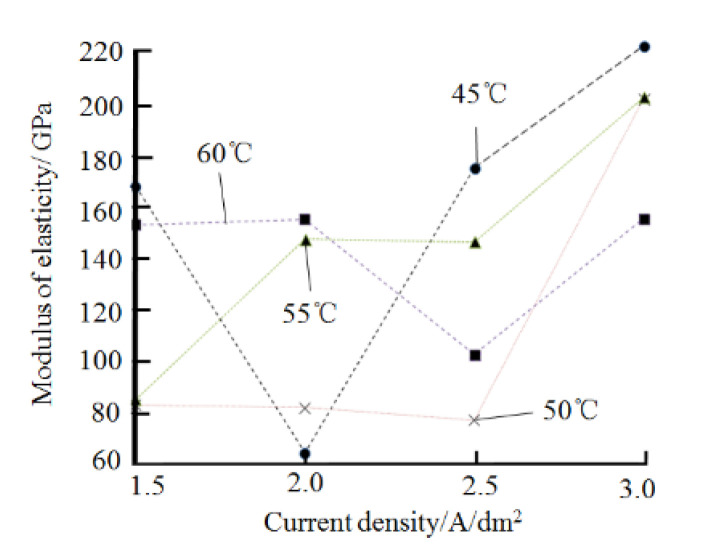
Curves of the relationship between current density, bath temperature, and the elastic modulus of the plated layer.

The effect of the pH of the plating solution on the elastic modulus of the plated layer is shown in Figure 8c. As the pH of the plating solution increases, reaction products Ni(OH)_2_ is generated near the cathode and entrapped in the plating layer, resulting in coarse grains in the plating layer and a decrease in the density of the plating layer, thus reducing the elastic modulus of the plating layer. However, when the pH of the plating solution continues to rise to 5, the elastic modulus of the plated layer increases significantly. The exact cause of this phenomenon needs to be further investigated. Figure 9 shows the relationship between the current density, the temperature of the plating solution, and the elastic modulus of the coating. According to Figure 9, in order to prepare nickel deposits with high modulus of elasticity by electrodeposition, the preferred plating process parameters are as follows: (1) current density at 2.5 A/dm^2^, bath temperature at 35 °C, pH 3.5 or pH 4.0; (2) current density at 3.5 A/dm^2^, bath temperature at 45 °C, pH 3.5 or pH 4.0.

#### 3.2.3. Effect on Surface Roughness of the Plated Layer

**Figure 10:**
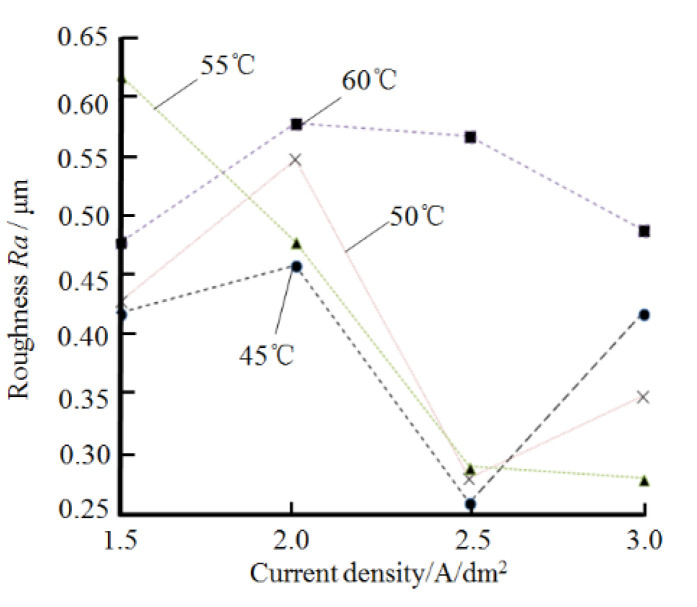
Effect of current density and bath temperature on the surface roughness Ra of the plated layer.

According to the analysis of the experimental results, when the pH value of the plating solution is between 3.5 and 5.0, the change of pH has less effect on the roughness of the surface of the plating layer. Figure 10 shows the relationship between current density, bath temperature, and the surface roughness Ra of the plated layer. According to Figure 10, in order to obtain a smooth surface nickel coating, the preferred plating process parameters are as follows: (1) current density at 2.5 A/dm^2^, plating solution temperature 45 °C ~55 °C; (2) current density at 3.0 A/dm^2^, plating solution temperature 50 °C~55 °C.

#### 3.2.4. Effect on Residual Stresses in the Plating

**Figure 11:**
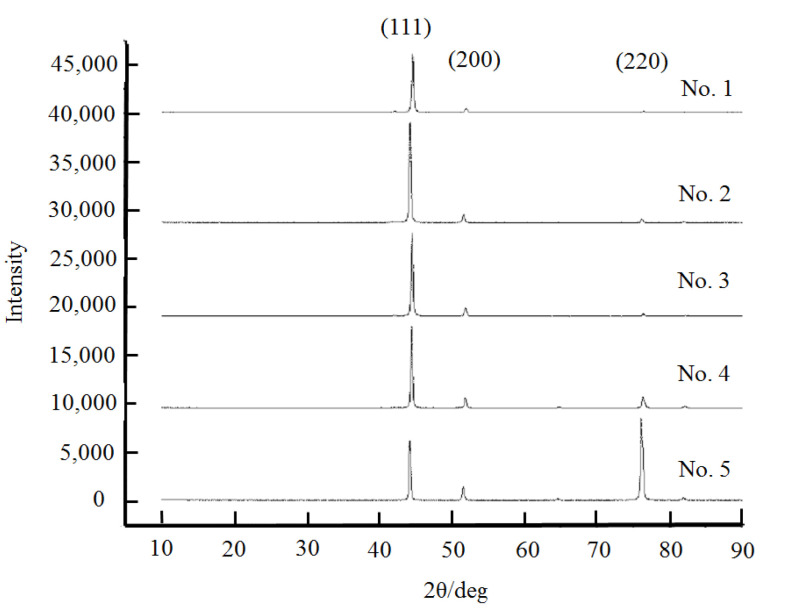
XRD diffraction patterns of five selected representative samples.

From the samples tested above, five representative specimens are selected for X-ray diffraction testing based on the hardness and elastic modulus of the plating. The effect of the plating process parameters on the selective orientation, residual stress, and grain size of the nickel-plated layers is analyzed. The XRD diffraction pattern of a representative five specimens is shown in Figure 11. According to Figure 11, the diffraction peaks of X-rays appear along the crystal faces (111), (200), and (220) during the testing of five selected representative specimens. From the bottom up, the diffraction peaks on each crystal plane become wider, indicating that the grain size of the coating becomes smaller; from the top downwards, the diffraction peaks on the crystalline plane (200) of the nickel plating gradually increase.

The specific conditions of the crystalline surface can be characterized by the texture coefficients of the crystalline surface. Based on the literature [21,23], the grain crystal surface texture coefficients and the grain size of the five selected representative samples here are calculated; the results are listed in Table 2. The calculations show that the residual stresses in the plated layers prepared under different plating conditions are distinct, with both compressive and tensile stresses, but the stresses are all below 81 MPa. The stress values are lower than the residual stresses in the plating studied by Kim et al. [24], with values between 160 and 220 MPa. The main reason for this is the use of agitation during electrodeposition, which prevents the hydrogen from adhering to the substrate surface and thus reduces the porosity of the prepared nickel plating. In addition, the thickness of the layer prepared by electrodeposition here is greater. At the beginning of electrodeposition, the crystal structure of the nickel plating layer is distorted in order to match the lattice of the substrate, and the residual stresses are high. However, as the thickness of the nickel plating increases, the crystal structure of the nickel plating gradually normalizes, so that the residual stresses within the prepared nickel plating are reduced.

**Figure 12:**
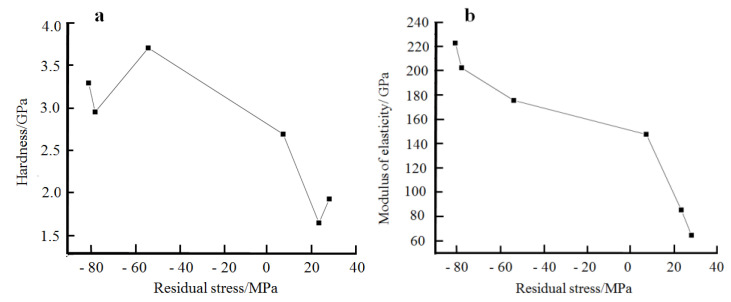
Curve of residual stress in the plating versus hardness and modulus of elasticity. (**a**) hardness, (**b**) Modulus of elasticity.

According to the calculations in Table 2, the residual stresses in the nickel plating show an increasing trend as the texture coefficients of the crystal plane (111) increases. AriGur et al. found in electroplated Ni-SiC composite coatings [25] that there was a close link between residual stresses and the specific orientation of the grain surface. The enhancement of the diffraction peaks along the crystalline surface (200) of the plating could indicate a significant reduction the residual stresses in the plating. But the increase in the texture coefficients of the crystalline surface (111) is caused by a selective oriented growth of the plating, making it possible for the plating to deviate from free growth, resulting in an increase in residual stresses inside the plating. This conclusion is consistent with the experimental results presented here. In addition, the hardness of the nickel deposits prepared by electroplating increases as the grain size decreases, a finding that is consistent with that of Hall-Petch. Based on the experimental results, it is found that there is a relationship between hardness, modulus of elasticity, and residual stress in the plating, as shown in Figure 12. The hardness and modulus of elasticity values are higher when the coating is under residual compressive stress. When residual tensile stresses are present in the coating, the hardness and modulus of elasticity values are smaller. At the same time, as the residual tensile stress increases, there is an overall tendency for the hardness and modulus of elasticity of the plating to decrease.

Bolshakov et al. [26] simulated load-displacement curves for plating with different residual stresses pressed into the same depth by using finite elements. The results of the simulations show that when the residual stress is compressive, the load required increases as the compressive stress increases; however, when the residual stress is tensile, the trend is the opposite. The simulation results are consistent with our experimental results here. Calculations using the Oliver and Pharr method [27] are performed as follows:

(3) $$H=P_{\max}/A$$

(4) $$\frac{1}{E_{r}}=\frac{1-\nu^{2}}{E_{}}+\frac{1-\nu_{i}{}^{2}}{E_{i}}$$

where *H* and *E* are the microhardness and elastic modulus of the coating, respectively, *A* is the indentation area, *Er* is the approximate elastic modulus, *ν* is Poisson’s ratio of the coating, and *E_i_* and *ν_i_* are the elastic modulus and Poisson’s ratio of the diamond indenter, respectively. The indentation area *A* and the approximate modulus of elasticity *E_r_* are then determined by means of the maximum load *P*_max_, i.e.,

(5) $$h_{c}=h_{\max}-\varepsilon \frac{P_{\max}}{S}$$

(6) $$A=24.505h_{c}{}^{2}$$

(7) $$E_{r}=\frac{1}{\beta }\frac{\sqrt{\pi }}{2}\frac{S}{\sqrt{A}}$$

where *h_c_* is the true indentation depth of the material tested, *S* is the material stiffness, and *ε* and *β* are both constants. Therefore, the hardness and modulus of elasticity of the material decrease as the residual internal material stress increases.

Based on the analysis above, in order to obtain a high level of hardness for the plated layer, the best process parameters for nickel plating are chosen as follows: current density at around 2.5 A/dm^2^, plating solution temperature at 45 °C, pH 3.5. The optimal process parameters for nickel plating in order to achieve a high elastic modulus are as follows: current density of about 3 A/dm^2^, bath temperature of 45 °C, and pH 3.5. In order to achieve a smooth surface for the prepared nickel layer, the optimal process parameters for nickel plating are as follows: current density at around 3 A/dm^2^ and temperature at around 50 °C. To analyze the effect of plating process parameters on the texture coefficients, grain size, and residual stress of the nickel plated layer, as well as to consider the influence of current density, bath temperature, and pH on the hardness, elastic modulus, and surface roughness Ra of the nickel plated layer, the optimal plating process parameters are obtained as follows: current density at 3 A/dm^2^, bath temperature at 45 °C, and pH 3.5.

### 3.3. Validation Tests after Optimization of Plating Process Parameters

**Figure 13:**
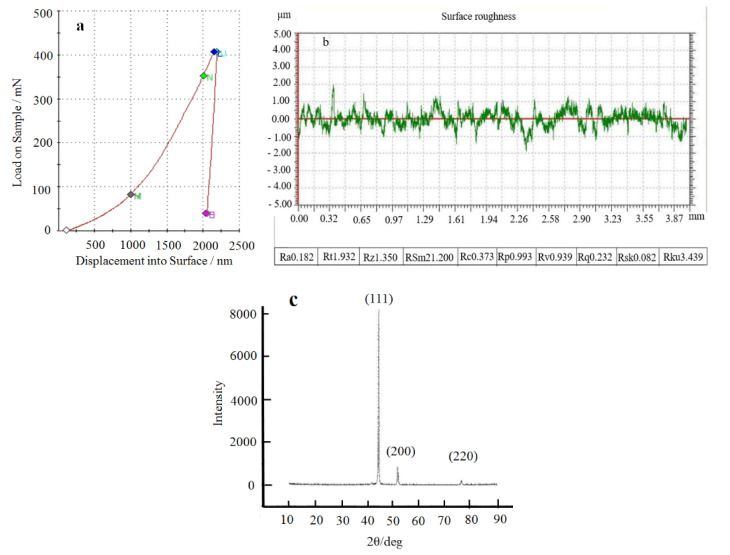
Tests on the properties of nickel plating prepared under the optimum process parameters scheme. (**a**) Load-displacement curve, (**b**) Ra, (**c**) X-diffraction pattern.

For this study, the nickel plating was prepared using the plating process parameters determined according to orthogonal tests. Figure 13 shows the test results for the preparation of nickel plating with the optimal plating process parameters. In particular, Figure 13a shows the load-displacement curve measured by the Nanoindentation Test System. After analysis and calculation, the microhardness and modulus of elasticity of the nickel plating were obtained at 3.86 GPa and 238 GPa, respectively. The result of the surface roughness parameter Ra for the nickel plating is shown in Figure 13b, at 0.182 μm. Figure 13c shows the X-ray diffraction pattern of the resulting nickel plating, and the display of the nickel plating along the (111) crystalline plane with a selective orientation. The grain size of the nickel coating calculated using Scherrer’s formula is 34.8 nm. High-strength nickel plating on mild steel Q235A surfaces was prepared using the optimal process parameter scheme with good test repeatability.

## 4. Conclusions

This paper explores the process parameters for obtaining the best mechanical properties of monolithic nickel layers on a Q235 substrate using the DC method with microhardness, modulus of elasticity, residual stress, and surface roughness as the optimization objectives. The results of the study show that under the process conditions of current density at 3 A/dm^2^, temperature at 45 °C, and pH 3.5, the mechanical properties of the resulting plating reaches the optimum. The minimum grain size, microhardness, modulus of elasticity, and surface roughness of the nickel plating are 34.8 nm, 3.86 GPa, 238 GPa, and 0.182 μm, respectively. The results presented here provide technical support for subsequent research into the preparation of high-strength nickel/nanodiamond composite coatings.

## Figures and Tables

**Table 1:** Table of Factor Levels for Orthogonal Tests.

Level	Current Density/A/dm^2^	Temperature/°C	pH
1	1.5	45	3.5
2	2.0	50	4.0
3	2.5	55	4.5
4	3.0	60	5.0

**Table 2:** Residual stress of Nickel coatings.

Specimen No.	Hardness/GPa	Residual Stress/MPa	Grain Size/nm	(111) Texture Coefficients
1	3.29	−80.74	28.6	77.92
2	2.95	−77.84	29.3	74.07
3	3.71	−53.78	32.1	73.30
4	1.93	28.11	35.2	52.05
5	1.65	23.43	39	12.40

## Data Availability

The data presented in this study are available on request from the corresponding author.
